# Characterization and Thermal Dehydration Kinetics of Highly Crystalline Mcallisterite, Synthesized at Low Temperatures

**DOI:** 10.1155/2014/985185

**Published:** 2014-02-25

**Authors:** Emek Moroydor Derun, Fatma Tugce Senberber

**Affiliations:** Department of Chemical Engineering, Yildiz Technical University, 34210 Istanbul, Turkey

## Abstract

The hydrothermal synthesis of a mcallisterite (Mg_2_(B_6_O_7_(OH)_6_)_2_·9(H_2_O)) mineral at low temperatures was characterized. For this purpose, several reaction temperatures (0–70°C) and reaction times (30–240 min) were studied. Synthesized minerals were subjected to X-ray diffraction (XRD), fourier transform infrared (FT-IR), and Raman spectroscopies and scanning electron microscopy (SEM). Additionally, experimental analyses of boron trioxide (B_2_O_3_) content and reaction yields were performed. Furthermore, thermal gravimetry and differential thermal analysis (TG/DTA) were used for the determination of thermal dehydration kinetics. According to the XRD results, mcallisterite, which has a powder diffraction file (pdf) number of “01-070-1902,” was formed under certain reaction parameters. Pure crystalline mcallisterite had diagnostic FT-IR and Raman vibration peaks and according to the SEM analysis, for the minerals which were synthesized at 60°C and 30 min of reaction time, particle size was between 398.30 and 700.06 nm. Its B_2_O_3_ content and reaction yield were 50.80 ± 1.12% and 85.80 ± 0.61%, respectively. Finally, average activation energies (conversion values (*α*) that were selected between 0.1 and 0.6) were calculated as 100.40 kJ/mol and 98.31 kJ/mol according to Ozawa and Kissinger-Akahira-Sunose (KAS) methods, respectively.

## 1. Introduction

Boron most often occurs in nature as borates which can be classified by the kind of metal it is complexed with. Magnesium borate minerals, which are a subclass of boron minerals, are inorganic compounds containing magnesium and boron. They are excellent additives for industry due to their high elasticity coefficient, heat resistance, and corrosion resistance [1]. Magnesium borates have specific applications in modified glass compositions, reinforcements in electronic ceramics, wide band gap semiconductors, aluminum/magnesium matrix alloys, antiwear additives such as thermoluminescence dosimeters, catalysts for the conversion of hydrocarbons, cathode ray tube screens, and X-ray screens [2–5].

Many kinds of magnesium borates having *x*MgO·*y*B_2_O_3_·*z*H_2_O compositions can be found naturally in mixture with other metal borates or can be obtained in the laboratory by synthetic methods. Some examples of this type of borate hydrate minerals that have been synthesized are 2MgO·3B_2_O_3_·17H_2_O, MgO·3B_2_O_3_·3.5H_2_O, 2MgO·B_2_O_3_·H_2_O, 2MgO·6B_2_O_3_·15H_2_O, and MgO·3B_2_O_3_·7H_2_O [6–13]. Mcallisterite is a type of magnesium borate with the chemical formula Mg_2_[B_6_O_7_(OH)_6_]_2_·9H_2_O. It has the appearance of very fine aggregates and white-colorless crystals, hardness of 2.5 Mohs, and low water solubility. Mcallisterite reserves are found in Argentina, China, Kazakhstan, and USA; however, in these reserves, magnesium and calcium borates are found in a mixture and purification is needed [14]. General hydrothermal synthesis procedures for magnesium borates involve the reactions of suitable raw materials at high temperatures such as >100°C or by double salt phase transformation. The type of experimental procedure used has effects on the product's crystal properties and size.

In literature, there are some examples of materials' surface modification by changing the reaction temperatures and reaction times. According to these studies, nanoscale materials can be synthesized as different crystal types [6–13, 15].

Hydrothermal processes have several advantages over the other types of conventional synthesis processes such as solid-state method in regard to energy conservation, better nucleation control, and lower temperature and pressure of operation [16, 17]. Higher reaction temperatures and longer reaction times cause increases in process cost.

Dehydrations of crystalline solids represent an important group of heterogeneous reactions. Characteristic dehydration features of materials should be known in order to determine design parameters of equipment and to decrease mass of required materials, thus reducing the transportation costs. The decomposition process of the hydrated boron mineral, which usually involves dehydration and dehydroxylation, can be explained by the removal of crystal water from structure [5–19]. Dehydration behaviors of different types of metal borate minerals have been determined by thermogravimetric analyses such as TG/DTA [1].

The effects of different nonisothermal kinetic methods on the thermal dehydration of inderite were examined by Zhu et al. [7]. Changes in ulexite structure resulting from heating and the reaction kinetic parameters were studied by Ener et al. [20] and Tunç et al. [21]; their results showed that ulexite could be turned to amorphous phase of NaB_3_O_5_ at 855°C. Waclawska [22] studied the effect of mechanical treatment on phase transitions of calcium borate and colemanite and internal structure reconstitution processes of ground colemanite. There have also been some studies regarding dehydration kinetics of synthesized boron compounds. Kanturk et al. [23] studied dehydration kinetic parameters such as activation energy and preexponential factors of synthesized sodium metaborate tetrahydrate (NaB(OH)_4_·2H_2_O). Kinetic analyses of boric acid thermal decomposition were studied by thermogravimetric analysis and different kinds of nonisothermal kinetic methods were used for the calculation of parameters [24]. Guo et al. [25] have investigated the decomposition and oxidation behavior of MgB_2_ using TG, XRD, and SEM-EDS.

In literature, despite the extensively reported synthesis of magnesium borates, only inderite minerals' kinetic behavior has been studied. To date, there have been no studies regarding the kinetic behavior of mcallisterite.

In this study, the low temperature (0–70°C) synthesis of a specific kind of magnesium borate mineral, namely, mcallisterite, is aimed. Therefore, in literature, Derun et al. [1] studied the magnesium borates between 80 and 100°C and synthesized a specific kind of magnesium borate mineral, namely, admontite. The other aim of this study is to determine the kinetic parameters (activation energy and coefficient factor) of mcallisterite mineral which was not studied before, with both Ozawa [26] and KAS [27, 28] nonisothermal kinetic methods.

## 2. Materials and Methods

### 2.1. Synthesis of Mcallisterite

The raw materials used in synthesis were boric acid (H_3_BO_3_), which was provided from Kırka Boron Management Plant (ETi Mine Kırka Works) in Eskisehir, Turkey, and magnesium oxide (MgO), which was provided from Merck Chemicals. H_3_BO_3_ was crushed, grinded, and sieved and MgO was used as supplied.

**Figure 1:**
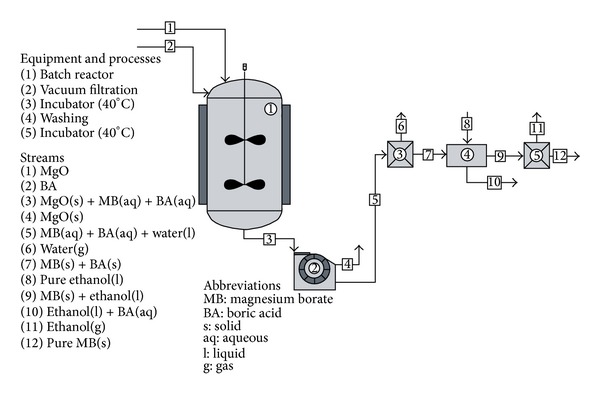
Synthesis procedure of magnesium borates.

The synthesis procedure of magnesium borates is given in Figure 1. Experiments were carried out at the reaction temperatures between 0 and 70°C and reaction time between 30 and 240 minutes. Each product was coded by initial letters of the raw materials (M: MgO and H: H_3_BO_3_), reaction temperature, and reaction time. For instance, “MH-60-30” indicated the product synthesized at a reaction temperature of 60°C and at a reaction time of 30 min.

### 2.2. Instrumental Analyses

Philips PANalytical XRD was used for identification of reaction products. X-rays were produced from a Cu-K*α* tube at 45 kV and 40 mA. The parameters used in the analyses were 0.030° step, 0.50 s time for step, 0.060°C/s scan speed, and 0–60° range. ICSD patterns were scanned using the inorganic library built into the instrument's program. Synthesized minerals were then subjected to FT-IR analyses using a Perkin Elmer FT-IR with universal attenuation total reflectance (ATR) sampling accessory with a diamond/ZnSe crystal. The measurement range was 1800–650 cm^−1^, scan number was 4, and resolution was 4 cm^−1^. For further analysis, Perkin Elmer Brand Raman Station 400 F was used for Raman spectroscopy. In these analyses, the exposure time was 4 seconds and the number of exposures was 4. Measurement range was 1800–250 cm^−1^ and the data interval was 2 cm^−1^. During the experiments, 100% laser power was used. Surface morphologies of synthesized minerals were obtained using a CamScan Apollo 300 field-emission SEM (20 kV and magnification 20000).

### 2.3. B_**2**_O_**3**_ Analyses and Reaction Yields

Both B_2_O_3_ analyses and calculations of reaction yields were performed according to Derun et al. [1].

### 2.4. Thermal Dehydration Kinetics

Thermal dehydration behavior of highly crystalline pure mcallisterite was studied between the temperature ranges of 20 and 720°C with a Perkin Elmer Diamond TG/DTA. Purely obtained mcallisterite mineral was subjected to five different heating rates (2°C/min, 5°C/min, 10°C/min, 15°C/min, and 20°C/min) in an inert (nitrogen) atmosphere. Kinetic parameters such as activation energy (*E*_*a*_) and coefficient constants (*k*_0_) were calculated by Ozawa and KAS nonisothermal kinetic methods.

In the Ozawa kinetic method (1), values of 1/*T* are plotted against log⁡*β* for each conversion value (*α*), where *T* is the thermodynamic temperature and *β* is heating rate. Activation energy (*E*_*a*_) is calculated from the slope of parallel lines. *R* is the gas constant. Consider

(1) $$\begin{array}{c} \log\beta =\log\left(\frac{k_{0}E_{a}}{R}\right)-2.315-0.4567\left(\frac{E_{a}}{RT}\right)-\log\left(g\left(\alpha \right)\right). \end{array}$$

In the KAS kinetic method (2), the kinetic parameters are determined from the plot of 1/*T* against the left side of equation for each *α* value:

(2) $$\begin{array}{c} \ln\left(\frac{\beta }{T^{2}}\right)=\ln\left(\frac{k_{0}E_{a}}{R\cdot g\left(x\right)}\right)-\frac{E_{a}}{RT}. \end{array}$$

### 2.5. Thermal Conversion of Mcallisterite

In order to investigate and characterize the product obtained after the thermal dehydration kinetics study, mcallisterite mineral was placed in a Protherm MOS 180/4 high temperature furnace with 10°C/min temperature increment to a maximum temperature of 720°C in nitrogen flowing (5 mL/min) atmosphere. After the thermal conversion, the product was analyzed by XRD with the same parameters given in Section 2.2.

## 3. Results and Discussion

### 3.1. XRD Results

The magnesium and boron sources used in the experiments were found to be periclase [MgO] and sassolite [H_3_BO_3_] with powder diffraction file (pdf) numbers of 01-087-0651 and 01-073-2158, respectively.

Products of the synthesis were determined to be mcallisterite [Mg_2_(B_6_O_7_(OH)_6_)_2_·9H_2_O] (pdf 01-070-1902), admontite [MgO(B_2_O_3_)_3_·7H_2_O] (pdf 01-076-0540), and magnesium borate hydrate [MgB_6_O_7_(OH)_6_·3(H_2_O)] (pdf 01-073-0638).

XRD scores of synthesized minerals, where a perfect crystal structure is equal to 100, are given in Table 1. MH-0-60, MH-10-30, MH-60-30, and MH-70-60 were pure mcallisterite. MH-20-120, MH-20-240, MH-30-240, and MH-40-240 were pure admontite. MH-70-240 was a mixture of three types of magnesium borate hydrate minerals.

**Figure 2:**
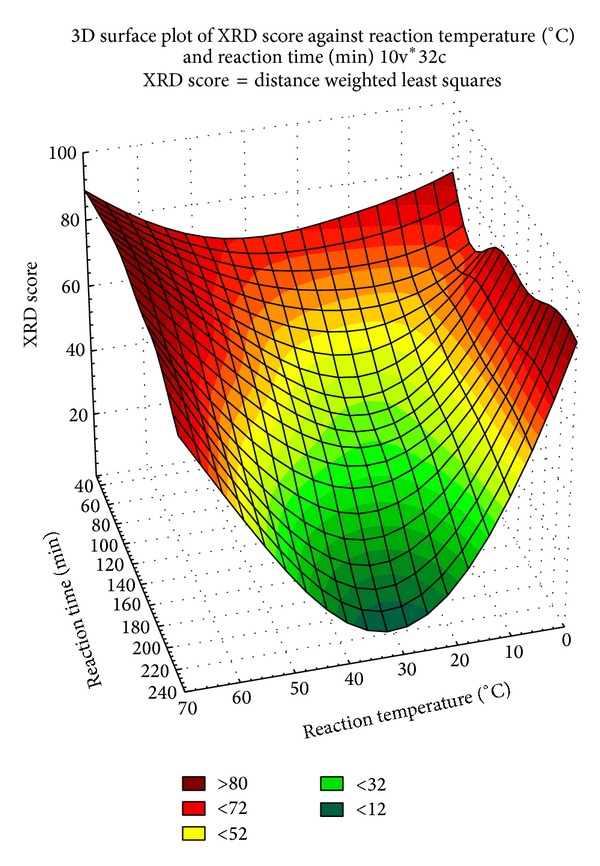
Modeling graph of mcallisterite crystal scores, which is drawn using Statsoft Statistica.

Mcallisterite formation as a function of reaction temperature and reaction time is presented in Figure 2. Mcallisterite crystal formation decreased from 0°C to 30°C and increased from 30°C to 70°C. Also, mcallisterite formation had a general tendency to increase with decreasing reaction times except at the temperatures of 0°C, 30°C, 50°C, and 60°C. At 0°C and 50°C, the maximum formation was seen at 120 min, whereas at 30°C and 60°C, the maximum formation was seen at 60 min.

The highest mcallisterite crystal scores were seen in MH-60-30 and MH-70-60 with values of 84 and 85, respectively. Since the XRD crystal scores for MH-60-30 and MH-70-60 were approximately the same, according to green chemistry concepts, MH-60-30 was selected as the best reaction parameter and subjected to TG/DTA kinetic analyses.

**Figure 3:**
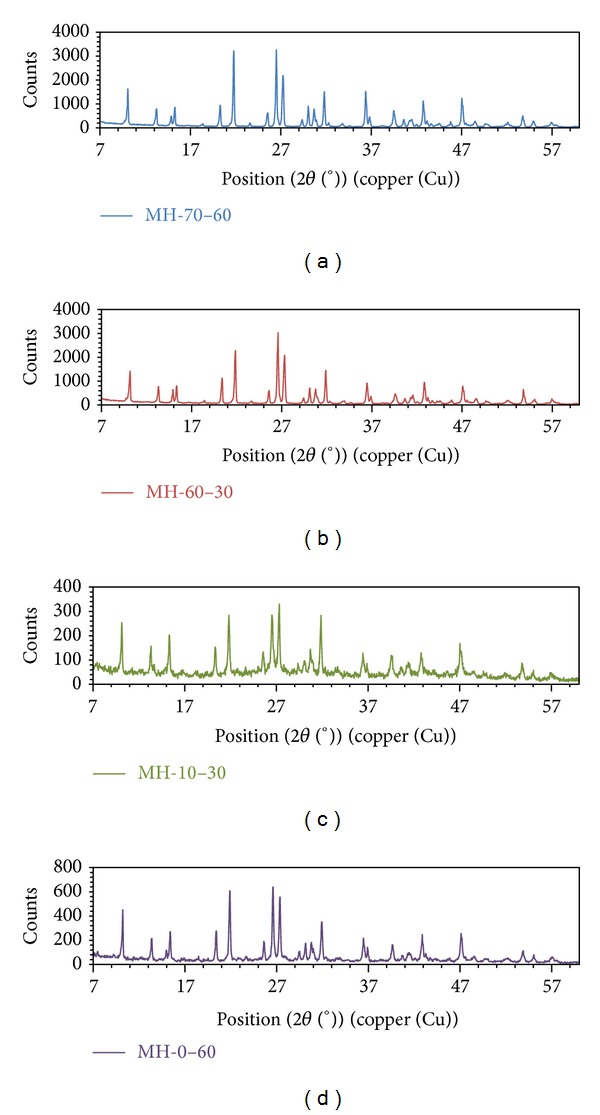
XRD patterns of synthesized pure mcallisterite minerals.

XRD patterns of synthesized pure mcallisterite minerals are given in Figure 3. As seen in Figure 3, all the characteristic peaks of mcallisterite were seen and higher count values were observed for MH-60-30 and MH-70-60 which is consistent with their higher crystal scores.

### 3.2. FT-IR and Raman Spectrum Results

**Figure 4:**
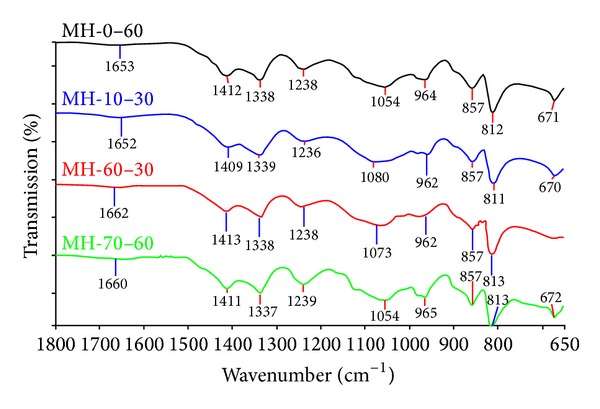
FT-IR spectrum of synthesized pure mcallisterite minerals.

FT-IR spectrum of product is given in Figure 4. The first peak at about 1650–1660 cm^−1^ is the bending of H–O–H [*δ*(H–O–H)]. The peaks at 1412–1337 cm^−1^ can be explained by asymmetric stretching of 3-coordinate boron [*υ*_as_(B_(3)_−O)]. The peak around 1238 cm^−1^ represents the bending of B–O–H [*δ*(B–O–H)]. Asymmetric and symmetric stretching of 4-coordinate boron [*υ*_as_(B_(4)_−O)], [*υ*_s_(B_(4)_−O)] were seen between the peaks of 1080–961 cm^−1^ and 857–812 cm^−1^, respectively. The last peak of 671 cm^−1^ was the bending of 3-coordinate boron [*δ*(B_(3)_−O)].

**Figure 5:**
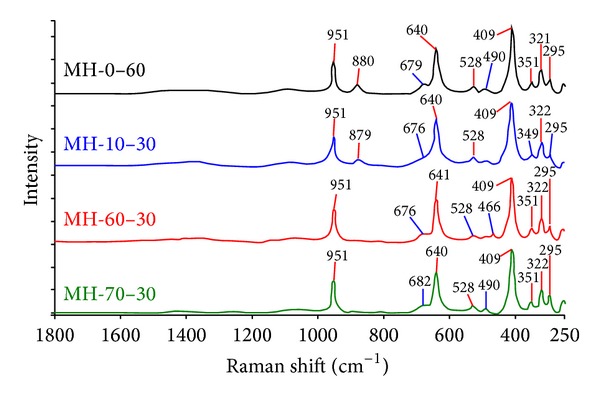
Raman spectrum of synthesized pure mcallisterite minerals.

Raman spectrum of the pure mcallisterite minerals is given in Figure 5. From the Raman results, symmetric stretching of 3-coordinate boron [*υ*_s_(B_(3)_−O)] was seen at the peaks between 951 and 879 cm^−1^. *δ*(B_(3)_−O) was seen at the peaks between 680 and 678 cm^−1^. The characteristic peaks of magnesium borates, which are *υ*_*p*_[B_6_O_7_(OH)_6_]^2−^ and *υ*_*p*_ [B_3_O_3_(OH)_4_]^−^, were seen at the peak values around 640 cm^−1^. At the peak of 528 cm^−1^, *δ*(B_(3)_−O) and bending of 4-coordinate boron [*δ*(B_(4)_−O)] were seen. The last peaks which are lower than the 490 cm^−1^ can be explained by the *δ*(B_(4)_−O).

The FT-IR and Raman results are both consistent with the literature [29, 30].

### 3.3. SEM Results

**Figure 6:**
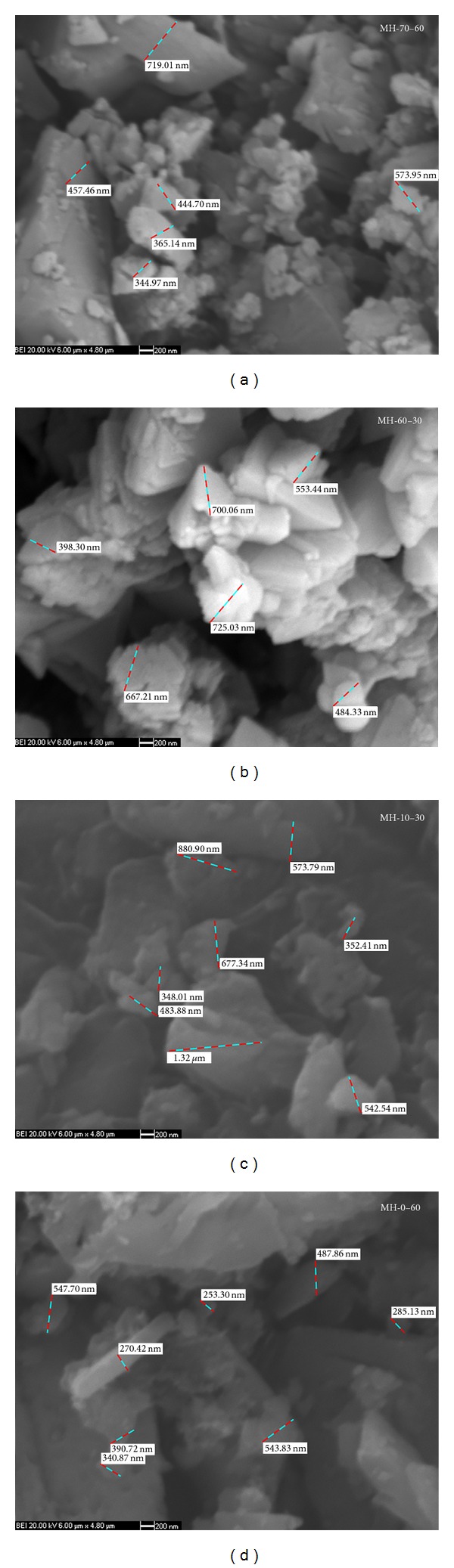
SEM surface morphologies of synthesized pure mcallisterite minerals at 20000x magnification.

SEM surface morphologies of the synthesized pure mcallisterite minerals are given in Figure 6. At 10°C and 0°C, crystals were seen as rectangular shapes due to overlapping of layers and single crystals. Particle sizes of the crystals at 10°C and 0°C were between 348 nm–1.32 *μ*m and 285–544 nm, respectively. Cylindrical crystal formations occurred at 60°C and 70°C, where particle sizes were 344–719 nm and 398–700 nm, respectively.

### 3.4. B_**2**_O_**3**_ Results and Reaction Yields

B_2_O_3_ contents of the synthesized minerals are given in Table 2. Highest and lowest B_2_O_3_ were seen in MH-50-30 (51.62 ± 1.07%) and MH-0-30 (44.59 ± 1.34%). Pure mcallisterite minerals B_2_O_3_ contents were 53.25 ± 1.20% in MH-70-60, 54.17 ± 0.87% in MH-60-30, 49.91 ± 1.28% in MH-10-30, and 45.92 ± 0.54% in MH-0-60. These results were in mutual agreement with theoretical B_2_O_3_ content of mcallisterite mineral (54.35%).

Average reaction yield of the MH-60-30 was 85.80 ± 0.61% as calculated from the four repeated syntheses.

### 3.5. Kinetic Analysis Results

**Figure 7:**
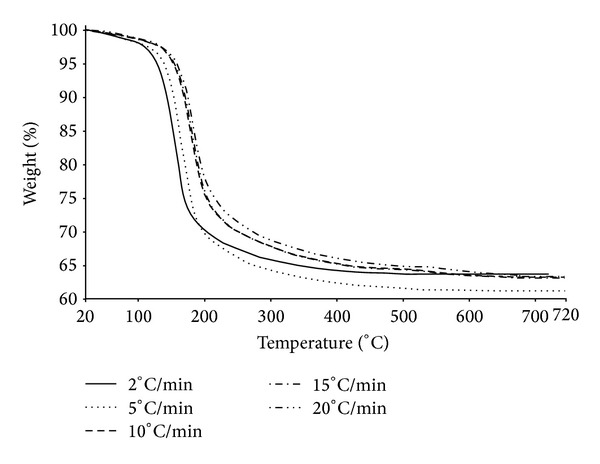
TG curve of synthesized pure mcallisterite.

**Figure 8:**
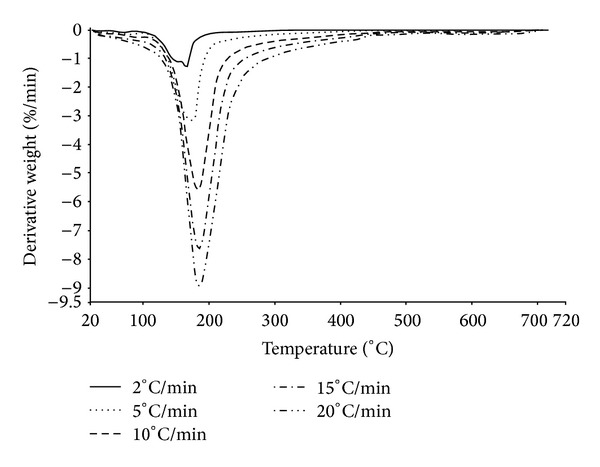
DTG curve of synthesized pure mcallisterite.

TG and DTG analyses of MH-60-30 are shown in Figures 7 and 8, respectively. The analyses showed that mcallisterite lost its crystal water via a two-step process at the heating rate of 2°C/min and by a single-step process at heating rates of greater than 2°C/min (5°C/min, 10°C/min, 15°C/min, and 20°C/min).

The first step at the heating rate of 2°C/min was a rapid dehydration, where the initial, peak, and final temperatures were 90.81°C, 150.64°C, and 155.94°C, respectively. In the second step, initial, peak, and final temperatures were 155.94°C, 165.79°C, and 300.00°C. Weight decreases were 16.416% and 19.359% for the first and second steps, respectively. Total weight loss was 35.775%.

The initial, peak, and final temperatures and weight losses at other heating rates are given in Table 3. The average weight loss, calculated using all of the heating rates, was 35.379%, which is close to structural water content (35.16%) of mcallisterite mineral.

**Figure 9:**
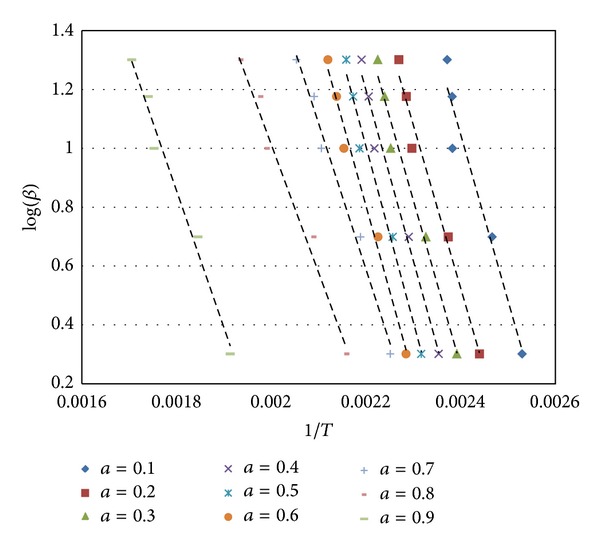
Ozawa analysis of mcallisterite.

Ozawa and KAS nonisothermal kinetic methods were applied for conversion values (*α*) between 0.1 and 0.9. In the Ozawa kinetic method, log⁡(*β*) values were plotted against 1/*T* values for each *α* value (Figure 9). For each heating rate kinetic parameter of *E*_*a*_ was calculated from the slope of the curves.

**Figure 10:**
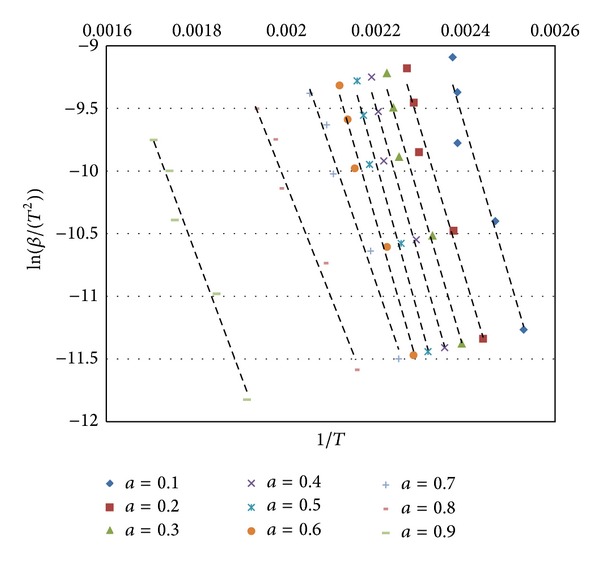
KAS analysis of mcallisterite.

Likewise, in the KAS kinetic method, ln⁡(*β*/*T* ^2^) was plotted against 1/*T* for each *α* value (Figure 10). Kinetic parameters of *E*_*a*_ and *k*_0_ for each heating rate were calculated from the intercept and slopes of the curves, respectively.

*E*_*a*_, *k*_0_, and the correlation coefficient (*R* ^2^) values obtained for each curve are shown in Table 4.

The activation energy values were calculated as 47.81–101.18 kJ/mol and 53.91–103.95 kJ/mol according to Ozawa and KAS methods, respectively. *k*_0_ values were between 0.0002 and 2913.89 according to KAS.

Average activation energies of mcallisterite mineral calculated for the conversion values between 0.1 and 0.6 were 100.40 kJ/mol and 98.31 kJ/mol according to Ozawa and KAS, respectively.

### 3.6. Thermal Conversion Results of Mcallisterite

Thermal conversion results showed that mcallisterite mineral lost 35.74 ± 0.32% of its weight. This was in agreement with the TG analyses and mcallisterite's theoretical structural water content of 35.16%, which is equal to 15 molar equivalent of water.

Also, XRD analysis showed that the mcallisterite mineral had lost all of its structure water and changed to dehydrated magnesium minerals Mg(B_2_O_3_)_2_ (pdf 01-076-0666) and B_2_O_3_ (pdf 01-072-0626). The obtained Mg(B_2_O_3_)_2_ and B_2_O_3_ crystal scores were 71 and 24, respectively. At this step, in order to obtain pure Mg(B_2_O_3_)_2_, the mixture was washed with pure ethanol and then filtered and dried at 40°C. Dried mineral was again subjected to XRD analyses and found as the same dehydrated magnesium mineral Mg(B_2_O_3_)_2_ with a crystal score of 83. The increase in the crystal score means that the excess B_2_O_3_ content was removed and pure Mg(B_2_O_3_)_2_ was obtained. Also, according to the weight changes, before and after the washing step, Mg(B_2_O_3_)_2_ and B_2_O_3_ were found to be equimolar.

**Figure 11:**
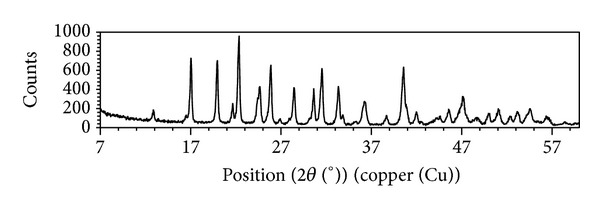
XRD pattern of Mg(B_2_O_3_)_2_.

The crystallographic data obtained from XRD are shown in Table 5 for mcallisterite and Mg(B_2_O_3_)_2_. The Mg(B_2_O_3_)_2_ XRD pattern is given in Figure 11, where in Figure 11 all the characteristic peaks of Mg(B_2_O_3_)_2_ were matched.

## 4. Conclusions

From the results of this study, it is seen that the pure mcallisterite minerals can be synthesized at a reaction temperature of 60°C with a 30 min reaction time by a hydrothermal method from the raw materials of MgO and H_3_BO_3_.

According to the XRD results, “01-070-1902” coded mcallisterite mineral [Mg_2_(B_6_O_7_(OH)_6_)·9H_2_O] was synthesized. FT-IR and Raman spectrum had the characteristic bands of magnesium borates [29, 30]. Surface morphologies revealed that proper crystals in nanoscale were obtained with particle size ranges of 398.30–700.06 nm. The B_2_O_3_ content of the MH-60-30 was 54.17 ± 0.87%, which is very close to the theoretical value of mcallisterite (54.35%). The average reaction yield of MH-60-30 was 85.80 ± 0.61%.

In thermal analysis at 2°C/min, mcallisterite lost its structure water content in a two-step process with the reaction scheme shown in (3) and (4):

 1st step:
  (3) Mg2(B6O7(OH)6)2·9H2O  ⟶Mg2(B6O7(OH)6)2·2H2O+~7H2O
 2nd step:
  (4) Mg2(B6O7(OH)6)2·2H2O  ⟶2(MgO(B2O3)2)+2B2O3+~8H2O

In the first step, mcallisterite lost approximately 7 moles of its structure water and in the second step the remaining 8 moles of structural water were lost. According to the thermal conversion results, the final components were equimolar Mg(B_2_O_3_)_2_ and B_2_O_3_.

In the thermal analyses at heating rates greater than 2°C/min, mcallisterite lost all 15 moles of structure water content in a single step, turning into Mg(B_2_O_3_)_2_ and B_2_O_3_ by the reaction scheme shown in (5):

 1st step:
  (5) Mg2(B6O7(OH)6)2·9H2O  ⟶2(MgO(B2O3)2)+2B2O3+15H2O

In the kinetic study, for the conversion values between 0.1 and 0.6, *R* ^2^ values varied in the range of 0.9909–0.9869 and 0.990–0.9849 in Ozawa and KAS method, respectively. Average *E*_*a*_ values of Ozawa and KAS methods were calculated as 100.40 KJ/mol and 98.31 KJ/mol, respectively.

In conclusion, the kinetic study of mcallisterite was reasonable considering that the Ozawa and KAS methods activation energy values were approximately the same.

## Figures and Tables

**Table 1:** XRD results of the synthesized magnesium borate minerals.

Reaction temperature (°C)	Reaction time (min)	XRD scores of		
		01-070-1902	01-076-0540	01-073-0638
0	30	78	30	—
	60	80	—	—
	120	84	25	—
	240	76	45	—

10	30	72	—	—
	60	8	6	—
	120	69	61	—
	240	76	45	—

20	30	85	31	—
	60	77	51	—
	120	—	71	—
	240	—	73	—

30	30	77	70	—
	60	84	71	—
	120	50	79	—
	240	—	81	—

40	30	52	81	—
	60	29	80	—
	120	39	80	—
	240	—	81	—

50	30	66	78	—
	60	86	59	—
	120	88	52	—
	240	45	79	—

60	30	84	—	—
	60	89	16	—
	120	85	63	—
	240	82	82	—

70	30	87	18	—
	60	85	—	—
	120	85	56	—
	240	57	84	23

pdf number = 01-070-1902, mcallisterite, Mg_2_(B_6_O_7_(OH)_6_)_2_·9(H_2_O).

pdf number = 01-076-0540, admontite, MgO(B_2_O_3_)_3_·7(H_2_O).

pdf number = 01-073-0638, MgB_6_O_7_(OH)_6_·3(H_2_O).

**Table 2:** B_2_O_3_ contents (%) of the synthesized magnesium borate minerals.

Reaction temperature (°C)	Reaction time (min)	B_2_O_3_ content (%)
0	30	44.58 ± 1.34
	60	45.92 ± 0.54
	120	47.06 ± 0.54
	240	45.73 ± 0.27

10	30	49.91 ± 1.28
	60	47.06 ± 0.54
	120	45.92 ± 0.54
	240	47.63 ± 0.81

20	30	47.82 ± 1.07
	60	49.53 ± 0.27
	120	49.72 ± 0.54
	240	48.01 ± 0.81

30	30	45.54 ± 1.15
	60	47.54 ± 0.13
	120	46.11 ± 0.81
	240	48.20 ± 1.68

40	30	48.96 ± 1.07
	60	48.20 ± 1.07
	120	47.06 ± 0.54
	240	46.68 ± 1.61

50	30	51.62 ± 1.07
	60	48.96 ± 1.07
	120	50.86 ± 0.54
	240	51.43 ± 0.81

60	30	54.17 ± 0.87
	60	52.87 ± 1.25
	120	53.18 ± 0.94
	240	50.17 ± 1.24

70	30	50.83 ± 0.84
	60	53.25 ± 1.20
	120	49.62 ± 1.06
	240	53.84 ± 1.34

**Table 3:** Dehydration temperatures and weight losses of pure mcallisterite (MH-60-30).

Heating rate (°C/min)	2		5	10	15	20
Step	1st	2nd	1st	1st	1st	1st

*T*_*i*_ (^o^C)	90.81	155.94	100.00	106.51	111.88	119.74
*T*_*p*_ (^o^C)	150.64	165.79	172.14	182.94	184.86	186.22
*T*_*f*_ (^o^C)	155.94	300.00	347.79	394.36	396.80	497.95
Δ*m* (%)	16.416	19.359	35.109	35.537	35.517	34.958
ΣΔ*m* (%)	35.775		35.109	35.537	35.517	34.958
AverageΔ*m* (%)	35.379					

*i*: initial; *p*: peak; *f*: final; *m*: weight.

**Table 4:** Calculated kinetic parameters for KAS and Ozawa method.

*α*	Method				
	Ozawa		KAS		
	*E*_*a*_ (kJ/mol)	*R* ^2^	*E*_*a*_ (kJ/mol)	*k*_0_	*R* ^2^
0.1	97.49	0.9909	95.58	2913.89	0.9896
0.2	98.42	0.9888	96.38	1695.83	0.9872
0.3	100.02	0.9871	97.95	1592.02	0.9852
0.4	102.24	0.9869	100.18	1899.34	0.9849
0.5	103.95	0.9889	101.87	1965.44	0.9872
0.6	100.26	0.9914	97.88	470.80	0.9900
0.7	83.07	0.9794	79.64	3.0520	0.9750
0.8	64.07	0.9736	59.32	0.0106	0.9656
0.9	53.91	0.9674	47.81	0.0002	0.9533

*α*	Average *E*_*a*_ (kJ/mol)		Average *E*_*a*_ (kJ/mol)		
0.1–0.6	100.40			98.31	

**Table 5:** Crystallographic data of synthesized mcallisterite and MgO(B_2_O_3_)_2_.

Mineral name	Mcallisterite	Magnesium borate
pdf number	01-070-1902	01-076-0666
Chemical formula	Mg_2_(B_6_O_7_(OH)_6_)_2_·9(H_2_O)	MgO(B_2_O_3_)_2_
Molecular weight (g/mole)	768.56	179.55
Crystal system	Rhombohedral	Orthorhombic
Space group	Pr3c (No. 167)	Pbca (No. 61)
*a* (Å)	11.5490	13.7300
*b* (Å)	11.5490	7.9700
*c* (Å)	35.5670	8.6200
*α* (°)	90.00	90.00
*β* (°)	90.00	90.00
*γ* (°)	120.00	90.00
*z*	6.00	8.00
Density (calculated) (g·cm^−3^)	1.86	2.53
Characteristic peaks *I* (%)/2*θ* (°)	100.0/10.139	100.0/22.291
	35.7/15.332	94.4/17.033
	32.9/31.875	80.9/19.921
